# Production and characterisation of monoclonal antibodies against RAI3 and its expression in human breast cancer

**DOI:** 10.1186/1471-2407-9-200

**Published:** 2009-06-24

**Authors:** Hannah Jörißen, Nuran Bektas, Edgar Dahl, Arndt Hartmann, Anette ten Haaf, Stefano Di Fiore, Hans Kiefer, Andreas Thess, Stefan Barth, Torsten Klockenbring

**Affiliations:** 1 Fraunhofer IME, Department of Pharmaceutical Product Development, Aachen, Germany; 2 Molecular Oncology Group, Institute of Pathology, University Hospital of the RWTH Aachen, Aachen, Germany; 3 Department of Pathology, University Hospital Erlangen, Erlangen, Germany; 4 Institute for Molecular Biotechnology, RWTH Aachen, Aachen, Germany; 5 M-fold Biotech, Tübingen, Germany; 6 Current address: CureVac GmbH, Tübingen, Germany; 7 Department for Experimental Medicine and Immunotherapy, University Hospital of the RWTH Aachen, Aachen, Germany

## Abstract

**Background:**

RAI3 is an orphan G-protein coupled receptor (GPCR) that has been associated with malignancy and may play a role in the proliferation of breast cancer cells. Although its exact function in normal and malignant cells remains unclear and evidence supporting its role in oncogenesis is controversial, its abundant expression on the surface of cancer cells would make it an interesting target for the development of antibody-based therapeutics. To investigate the link with cancer and provide more evidence for its role, we carried out a systematic analysis of RAI3 expression in a large set of human breast cancer specimens.

**Methods:**

We expressed recombinant human RAI3 in bacteria and reconstituted the purified protein in liposomes to raise monoclonal antibodies using classical hybridoma techniques. The specific binding activity of the antibodies was confirmed by enzyme-linked immunosorbent assay (ELISA), western blot and immunocytochemistry. We carried out a systematic immunohistochemical analysis of RAI3 expression in human invasive breast carcinomas (n = 147) and normal breast tissues (n = 44) using a tissue microarray. In addition, a cDNA dot blot hybridisation assay was used to investigate a set of matched normal and cancerous breast tissue specimens (n = 50) as well as lymph node metastases (n = 3) for *RAI3 *mRNA expression.

**Results:**

The anti-RAI3 monoclonal antibodies bound to recombinant human RAI3 protein with high specificity and affinity, as shown by ELISA, western blot and ICC. The cDNA dot blot and immunohistochemical experiments showed that both *RAI3 *mRNA and RAI3 protein were abundantly expressed in human breast carcinoma. However, there was no association between RAI3 protein expression and prognosis based on overall and recurrence-free survival.

**Conclusion:**

We have generated a novel, highly-specific monoclonal antibody that detects RAI3 in formaldehyde-fixed paraffin-embedded tissue. This is the first study to report a systematic analysis of RAI3 expression in normal and cancerous human breast tissue at both the mRNA and protein levels.

## Background

RAI3 belongs to the family of G-protein coupled receptors (GPCRs) that are the largest and most abundant receptor family in mammals consisting of more than a thousand members. They are characterised by the characteristic structure of an extracellular ligand-binding domain, and internal seven-pass transmembrane domain (7-TM) consisting of seven membrane-spanning α-helices, and an internal C-terminal domain [1]. The intracellular C-terminus is thought to interact with G-proteins that bind guanidine-nucleotides (GDP, GTP) and can activate downstream effectors such as adenyl cyclases, phospholipases, phosphodiesterases and ion channels when an agonist binds to the extracellular portion of the receptor [2]. GPCRs activate numerous signal transduction cascades and thus play pivotal role in the regulation of many physiological and pathological processes, including cell growth and differentiation. For this reason, they are regarded as valuable and interesting drug targets for the numerous human diseases that have been associated with dysfunctional GPCRs, and researchers are actively seeking ligands for the so-called orphan GPCRs whose role in physiology and disease has yet to be determined [1]. The abundant cell surface expression and rapid internalisation of GPCRs make them particularly appropriate targets for antibody-based therapeutics.

*RAI3*, also known as *GPRC5A *and *RAIG-1*, was originally identified as an all-*trans*-retinoic acid inducible gene [3]. The corresponding orphan GPCR is localised at the plasma membrane and in intracellular vesicles, and is predominantly expressed in normal lung tissue whereas only low level expression has been detected in other tissues such as liver, pancreas, colon and mammary glands [3-5]. Although RAI3 is thought to regulate cell proliferation, the exact molecular mechanism is not understood and is a matter of some controversy [5-8]. *RAI3 *was initially considered to be a potential tumour-suppressor gene because of its presumed regulation by retinoids, which have shown to influence tumourigenesis and exert significant therapeutic and preventative effects on tumours. This hypothesis has recently been supported by data from a knock-out mouse model, which showed mice lacking the gene were more likely than wild type mice to develop lung tumours at 1–2 years of age [8]. RT-PCR analysis of different lung cancer types also showed higher *RAI3 *mRNA levels in normal tissue adjacent to tumour tissue. However, other findings indicate that higher levels of *RAI3 *expression promote tumour growth, e.g. a cDNA microarray study in which RAI3 was shown to be upregulated in breast cancer specimens [9]. In a further study, *RAI3 *overexpression was observed in 19 of 25 breast cancer samples compared to matched normal mammary gland tissues and in 6 of 11 breast cancer cell lines. In addition, siRNA silencing of *RAI3 *reduced proliferation of the human embryonic kidney cell line HEK293 and the breast cancer cell lines MCF-7 and T47D [5]. In *RAI3*-transfected Nthy cells, a human thyroid follicular epithelial cell line, *RAI3 *siRNA actually induced apoptosis [7]. Further studies have suggested *RAI3 *is a growth-promoting gene that may be involved in cell cycle regulation with a peak expression in G1 phase, as analysed in the human cervical epithelial tumour cell line (HeLa). The growth-promoting effect of RAI3 is further underlined by the finding that RAI3 is a p53-transcriptional target gene [6].

The role of RAI3 in cancer and its localisation in the plasma membrane make this molecule an interesting potential target for antibody-mediated therapy. We report for the first time the development of a monoclonal anti-human RAI3 antibody, termed Mab 24 2.3, that binds with high specificity and affinity to RAI3 in ELISAs, western blot and as well *in situ *to the target on transfected HEK293T cells and paraffin-embedded tissue sections. The development of this antibody is of general interest for potential diagnostic use in routine pathological investigations, but it could be used in a therapeutic context. As RAI3 was found upregulated in human breast cancer we decided to use the antibody to determine the abundance of RAI3 on a large panel of human breast carcinomas and normal breast tissues, and compare this with expression analysis at the mRNA level. This is the first systematic analysis of RAI3 expression in a large cohort of human breast carcinomas and normal breast tissues both at the mRNA and the protein level. We evaluated the results looking for correlations with hormone receptor status, HER2 status and patient survival data. The impact of our data on prognosis and treatment of breast cancer are discussed.

## Methods

### RAI3 antigen expression and purification

The *RAI3 *cDNA was cloned in pGEX2a as an in-frame fusion with an N-terminal GST tag and a C-terminal hexahistidine tag as described previously [10]. The N-terminal fusion abolishes the toxicity observed when eukaryotic membrane proteins are expressed in *E. coli *by promoting the direction of protein to inclusion bodies which results in high-level production [10]. The construct was introduced into *Escherichia coli *BL21, and 1 l of LB medium containing 100 mg/l ampicillin was inoculated with 10 ml of an overnight culture containing 50 mg/l carbenicillin and shaken at 37°C. When the culture reached an A_600 _of 0.80, protein expression was induced by adding 0.1 mM of isopropyl-β-D-thiogalactoside for 4 h. Cells were harvested by centrifugation, resuspended in 10 ml ice-cold 20 mM Tris/HCl pH 7.5 and frozen at -20°C. After thawing to 4°C and adding EDTA and dithiothreitol (DTT) each to a final concentration of 1 mM, the cell suspension was stirred for 10 min, disrupted by repeated passage through a high-pressure homogeniser (Emulsiflex C5, Avestin Inc. Canada), supplemented to a final concentration of 50 mM EDTA, 10 mg/ml Triton X-100, and stirred for a further 30 min. Insoluble protein was pelleted by centrifugation for 45 min at 48400 g. The pellet was washed in 20 mM sodium phosphate, 150 mM NaCl (pH 7.0) containing 1 mM DTT, centrifuged as above and finally resuspended in 10 ml of the same buffer. This suspension, referred to as "inclusion bodies", although it includes the bacterial membranes, was stored at -20°C.

Inclusion bodies were thawed on ice, pelleted by centrifugation for 30 min at 46500 g at 4°C, resuspended in 5 ml of 25 mM Tris/HCl pH 8.5, 150 mM NaCl and incubated at room temperature under reducing conditions (5 mM DTT) for 1 h. Ten volumes of 10 mg/ml lauroyl sarcosine (LS) in 25 mM Tris/HCl (pH 8.5), 250 mM NaCl were added and the sample was sonicated for 1 min using a tip sonicator on ice. After stirring at room temperature for 2 h and at 4°C for 1 h, the C-terminal GST tag was cleaved with 10 U thrombin per ml of inclusion bodies for 1 h at 4°C. To abort cleavage, we added 200 μM phenylmethylsulphonyl fluoride and an additional 10 mg/ml LS. The insoluble fraction was removed by centrifugation at 38500 g, 4°C. For RAI3 purification, the supernatant was incubated overnight at 4°C with 1.5 ml Ni-NTA Superflow (Qiagen, Hilden, Germany) per ml of inclusion bodies equilibrated in 20 mg/ml LS, 25 mM Tris/HCl (pH 8.5), 250 mM NaCl.

The Ni-matrix was packed into a column and washed at a flow rate of 2 cm/h with (i) three column volumes (CV) of 20 mM Tris/HCl (pH 8.5), 250 mM NaCl, 10 mM β-mercaptoethanol (βME), 10 mg/ml n-tetradecylphosphocholine (Fos14, Anatrace, Maumee, OH), (ii) 10 CV of 20 mM Tris/HCl (pH 8.5), 250 mM NaCl, 10 mM βME, 1 mg/ml Fos14, 0.2 mg/ml Folch Lipid Fraction I (Sigma) and (iii) one CV of 20 mM Tris/HCl (pH 7.5), 250 mM NaCl, 1 mM glutathione (GSH), 0.1 mg/ml Fos14. Protein was eluted in 20 mM Tris, 250 mM NaCl, 1 mM GSH, 300 mM imidazole, 0.1 mg/ml Fos14 adjusted to pH 7.5. Protein-containing fractions were pooled and DTT was added to 10 mM. Monomeric RAI3 was isolated by applying the concentrated pooled fraction to a Superdex 200 HiLoad XK 16/60 gel filtration column (GE Healthcare, Freiburg, Germany) run in 20 mM HEPES/NaOH (pH 7.0), 200 mM NaCl, 0.25 mg/ml Fos14, 1 mM DTT at 1 ml/min. Monomeric RAI3-containing fractions were pooled, and concentrated to 1 mg/ml.

### RAI3 reconstitution into liposomes

Purified RAI3 was dialysed against 100 volumes of 100 mM boric acid/NaOH (pH 9.5), 0.1 mg/ml Fos14 at 4°C prior to reconstitution. A lipid blend was obtained by dissolving Folch Lipid Fraction I, phosphatidyl ethanolamine from sheep brain (Sigma) and cholesterol at a ratio of 40:32:28 in chloroform. After removal of the chloroform under vacuum over night the lipid was first resuspended in water at 10 mg/ml and then partially dissolved in CHAPS by incubating for 1 h at room temperature with a CHAPS to lipid ratio of 3:1 and a lipid concentration of 7.5 mg/ml. CHAPS and SDS at final concentrations of 5 and 0.1 mg/ml respectively were added to the dialysed RAI3. After 10 min, 70 mM KCl was added and the RAI3 solution was merged with 1.3 times the volume of the lipid/CHAPS mixture. This mixture was rotated end-over-end over night at 4°C. Detergent was then extracted by adding an equal volume of fresh Calbiosorb beads, which had been thoroughly washed with water and drained, followed by end-over-end rotation for 3 h at 4°C. Beads were removed using a fritted plastic column and proteoliposomes were harvested by centrifugation for 30 min at 100,000 g, 4°C. The resulting pellet was resuspended in 20 mM HEPES/NaOH (pH 7.0), 150 mM NaCl at a theoretical protein concentration of 1 mg/ml. Aliquots were frozen in liquid nitrogen and stored at -80°C. BALB/c mice were immunised using the RAI3 antigen reconstituted in liposomes. All additional *in vitro *experiments were also performed with the soluble protein prior to liposome reconstitution.

### Production of monoclonal antibodies

Six-week-old female BALB/c mice were immunised subcutaneously with 100 μg RAI3 liposomes and 60 μl GERBU Adjuvant MM (GERBU Biochemicals, Gaiberg, Germany) as a priming injection following three booster injections with 50 μg RAI3 mixed with 40 μl adjuvant at 10-day intervals. Two days prior to fusion, a final booster injection was given comprising 50 μg RAI3 without adjuvant. On each day of immunisation a 10-μl blood sample was taken to monitor the specific antibody titre in the serum. The immune response was analysed by direct ELISA using plates coated with recombinant RAI3 protein (50 ng/well) and a HRP-conjugated anti-mouse IgG antibody (Sigma-Aldrich, Munich, Germany; 1:5,000) using a serial dilution of mouse serum.

On day 45, the spleen was dissected and spleen cells were fused with murine myeloma SP2-IL6 cells (ATCC: CRL-2016 [11]) at a ratio of 1:1 in the presence of PEG 1500 (Boehringer, Mannheim, Germany). Fused cells were cultured and selected in RPMI 1640 (Gibco Invitrogen, Carlsbad, USA) supplemented with 20% bovine calf serum (Biochrom AG, Berlin, Germany), 100 U/ml penicillin, 100 μg/ml streptomycin (Gibco Invitrogen), 100 μM hypoxanthine, 16 μM thymidine, and 0.4 μM aminopterin (Sigma-Aldrich), 80 U/ml murine interleukin-6 (Calbiochen, San Diego, USA) and 50 μM βME (Gibco Invitrogen) in 96-well microtitre plates. The selection medium was refreshed after one week. After 12 days, culture supernatants were screened for antibodies binding to RAI3 liposomes in a direct ELISA. Hybridomas in wells generating the highest ELISA signals were used for limiting-dilution cloning in HT medium (RPMI 1640 supplemented with 20% bovine calf serum, 100 U/ml penicillin, 100 μg/ml streptomycin, 100 μM hypoxanthine, 16 μM thymidine, 80 U/ml interleukin-6 and 50 μM βME). Monoclonal hybridomas were cultured in standard growth medium (RPMI 1640 supplemented with 10% bovine calf serum, 100 U/ml penicillin, 100 μg/ml streptomycin). Prior to purification, hybridomas were adapted to serum-free hybridoma culture medium ISF-1 (Biochrom AG). Antibodies were purified from culture supernatants by Protein-G affinity chromatography (ÄKTA_FPLC _systems, Amersham Biosciences, Freiburg, Germany). Isotypes were determined using a BD Bioscience (Heidelberg, Germany) isotyping kit (Heidelberg, Germany). Clone 24 2.3 was selected for immunohistochemical analysis.

### Direct ELISA for the analysis of hybridoma supernatants

Hybridoma supernatants containing monoclonal anti-RAI3 antibodies were analysed by direct ELISA. High-binding microtitre plates (Greiner Bio-One, Frickenhausen, Germany) were coated overnight at 4°C with varying amounts of recombinant RAI3 (0.2–200 ng/well) in 100 mM carbonate buffer (pH 9.5), or with 50 ng/well of the negative control proteins bovine serum albumin (BSA), an unrelated human protein (CD30 ligand), and a control GPCR (GPR30) produced under the same conditions as RAI3. Uncoated wells were used as an additional negative control. After blocking with 2% non-fat dry milk, 50 μl hybridoma supernatant was added directly to each well and incubated at room temperature for 1 h. Plates were washed three times with phosphate buffered saline containing 0.05% Tween20 (PBST). The binding of anti-RAI3 antibodies was detected with a HRP-conjugated anti-mouse antibody (Sigma-Aldrich; 1:5,000) and 2'-azino-bis(3-ethylbenzthiazoline-6-sulphonic acid) (ABTS) (Roche Diagnostics, Mannheim, Germany) as the substrate. Absorption was measured at 405 nm using an ELISA reader (Biotek Instruments, Bad Friedrichshall, Germany). Each measurement as performed in triplicate.

### Measuring binding affinity

The binding affinity of purified monoclonal antibodies was estimated in a competitive ELISA [12]. Briefly, high-binding microtitre plates were coated overnight at 4°C with 50 μl of the 1 μg/ml protein solutions described above. After blocking, a 1 nM antibody solution was pre-incubated with a serial dilution of RAI3 antigen in the molar range 2 × 10^-8^–2 × 10^-10^M until equilibrium was achieved. The antibody-antigen mixture was then applied to the coated microtiter plate in triplicate, to measure the unbound antibodies remaining in solution. The immobilised antibodies were detected and quantified as described for the direct ELISA. K_d _values were calculated from the absorption data as described elsewhere [12].

### Transfection of mammalian cells

RAI3 template DNA was kindly provided by M-Fold Biotech, Tübingen, Germany. The DNA was amplified with gene-specific primers containing appropriate restriction sites for cloning in pMS RAI3 III and pMS GFP-RAI3 MH, allowing RAI3 to be expressed under the CMV promoter with and without C-terminal GFP. The primers were NheI Kozak 5' (5'-cat tcg agG CTA G*cc acc *ATG GCT ACA ACA GTC CCT G-3'), NotI 3' (5'-act tgt caG CGG CCG CGC TGC CCT CTT TCT TTA CTT C-3') and SfiI 5' (5'-cat tcg agG GCC CAG CCG GCC ATG GCT ACA ACA GTC CCT G-3'). Restriction sites are underlined, small roman letters are anchor sequences and italic letters show the Kozak consensus. Mock expression vectors with and without GFP were generated as controls. HEK 293T cells (ATCC: CRL-11268) were cultured in RPMI 1640 supplemented with 10% bovine calf serum, 100 U/ml penicillin and 100 μg/ml streptomycin and transfected using Roti-fect (Carl Roth GmbH, Karlsruhe, Germany) according to the manufacturer's protocol. Transfected cells were selected by supplementing the medium with 100 μg/ml zeocin (Invitrogen).

### Western blot analysis of cell lysates

RAI3-transfected and mock-transfected HEK293T cells were harvested and agitated for 30 min at 4°C in a non-denaturing lysis buffer (20 mM Tris HCl (pH 8), 137 mM NaCl, 10% glycerol, 2 mM EDTA, 1% Triton X-100) containing a freshly added protease inhibitor cocktail tablet (Roche Diagnostics). The insoluble fraction was separated by centrifugation for 20 min at 12,000 × g. Cell lysates containing approximately 10 μg total protein were separated by sodium dodecylsulphate polyacrylamide gel electrophoresis (SDS-PAGE) on a 12% polyacrylamide gel and blotted to nitrocellulose membranes (Whatman Schleicher & Schuell, Dassel, Germany). Membranes were blocked for 1 h at room temperature in PBST containing 2% non-fat dry milk, washed three times in PBST and incubated with anti-RAI3 antibodies (100 ng/ml in PBST) overnight at 4°C. After three PBST washes, the membranes were incubated with HRP-conjugated anti-mouse antibody (Sigma-Aldrich; 1:5000 in PBST) for 1 h at room temperature, followed by three further washes. Specific binding was detected with ECL Western Blotting substrate (Pierce, Rockford, USA) and visualised in a LAS-3000 imager (Fujifilm). The blot was subsequently stripped by incubation in stripping buffer (62.5 mM Tris (pH 6.8), 2% SDS, 100 mM βME) for 45 min at 50°C. For loading control, the blot was reprobed with an HRP-conjugated anti-β-actin antibody (Sigma-Aldrich; 1:2000) and detected with the ECL system as described above.

### Immunocytochemistry

The specificity of the anti-RAI3 antibody (Mab 24 2.3) was further analysed by immunocytochemistry in HEK293T cells. Briefly, HEK293T cells transiently transfected with the expression vector encoding RAI3-GFP were seeded at 2.5 × 10^4 ^cells per well on a multi-well chamber slide (Nunc) and incubated overnight at 37°C to allow cells to attach. Cells were fixed in 4% paraformaldehyde, washed with PBS and subsequently permeabilised and blocked in PBS containing 0.5% Triton X-100 and 3% BSA. Following a washing step, the primary anti-RAI3 antibody Mab 24 2.3 was applied in a 1:100 dilution and incubated on the cells for 1 h at room temperature. Primary antibody binding was detected indirectly using a 1:2000 dilution of AlexaFluor-546 conjugated anti-mouse secondary antibody (A546; Invitrogen) which was incubated on the cells in the dark for 1 h at room temperature followed by a further washing step. The primary antibody was omitted in control cells. The cell nucleus was stained post fixation using a 20 μM Draq5 solution (Biostatus) in PBS followed by a final washing and a second fixation step. Images of the cells were acquired by confocal microscopy.

### Laser scanning confocal microscopy

Imaging was carried out by laser scanning confocal microscopy using 488, 561 and 635 nm excitation laser lines and tunable emission filters adjusted to cover emission ranges between 540/75, 600/40 and 685/70 nm for RAI3-GFP, AlexaFluor-546 and Draq5, respectively. Images of cells labelled with the three fluorophores were acquired using a 40× PlanApo air objective (0.85 numerical aperture) and sequential acquisition to prevent measuring "cross-talk" between the three emission signals. The images were pseudo coloured in Adobe Photoshop.

### Patient samples

RAI3 protein expression in breast cancer patients was assessed using a previously-described tissue microarray (TMA) [13] comprising 157 breast cancer specimens and 44 normal breast tissue samples. The TMA contained one tissue core from non-selected, formalin-fixed and paraffin-embedded primary breast cancer from patients aged 25–82 (median age of 56), diagnosed between 1994 and 2002 at the Institute of Pathology, University of Regensburg, Germany. An experienced surgical pathologist (A.H.) evaluated H&E-stained slides of all specimens prior to construction of the TMA in order to identify representative tumour areas. All tumours were graded according to Elston and Ellis [14]. Clinical follow-up data, provided by the Central Tumour Registry, Regensburg, Germany, were available for all 157 breast cancer patients with a median follow-up period of 78 months (range 0–148 months). All patients gave informed consent for retention and analysis of their tissue for research purposes and the Institutional Review Board of the participating centre approved the study.

### Breast cancer cDNA dot blot hybridisation

The breast cancer profiling array (CPA) (BD Clontech, Heidelberg, Germany) contains 50 pairs of cDNAs generated from matched cancerous and normal breast tissue samples of individual patients and three breast cancer lymph node metastasis specimens, spotted on a nylon membrane [15]. Hybridisations using 25 ng of a gene-specific ^32^P-labelled cDNA probe prepared from a RAI3 Unigene cDNA clone were carried out according to the manufacturer's recommendations. This hybridisation probe included base pair 550 to 2835 of the RAI3 cDNA deposited under accession number NM_003979. The tumour/normal intensity ratio was calculated using a STORM-860 phosphorimager (Molecular Dynamics, Sunnyvale, CA, USA) and normalised against the background.

### Immunohistochemical characterisation of the tissue microarray

Immunohistochemical studies of HER2 expression were carried out using the avidin-biotin peroxidase method with a 3,3'-diaminobenzidine (DAB) chromogen in a NEXES immunostainer (Ventana, Tucson, AZ, USA) following antigen retrieval by microwaving for 30 min. Primary anti-HER2 (DAKO, Hamburg, Germany; 1:400), anti-ER and anti-PR (Novocastra, Newcastle Upon Tyne, UK; 1:20) antibodies were detected using the ChemMate detection kit (DAKO). A surgical pathologist (A.H.) performed a blinded evaluation of the TMA slides without clinical data. Non-interpretable results were caused by a lack of tumour tissue and the presence of necrosis or crush artefacts. HER2 expression was scored according to the DAKO HercepTest. For the evaluation of ER and PR, a semi-quantitative immunoreactivity score (IRS) was used as described by Remmele and Stegner [16].

### RAI3 immunohistochemistry

The tissue microarray was stained using the Advance Kit (DAKO K5007) according to the manufacturer's instructions. Breast carcinomas were used as positive controls. After paraffin removal and rehydration, the tissue samples were boiled in a microwave oven for 30 min in 10 mM sodium citrate buffer (pH 7.6). Endogenous peroxidase was blocked by peroxidase blocking solution (DAKO S 2023) for 10 min. The anti-RAI3 antibody Mab 24 2.3, was selected for immunohistochemical application. It was applied at a dilution of 1:50 for 3–4 h at 4°C and detected with DAB. In negative controls the primary antibody was omitted. As an additional negative control, a competitive immunohistochemical analysis was performed by pre-incubation of the anti-RAI3 antibody Mab 24 2.3 with 200-fold molar excess of RAI3. Slides were counterstained with haematoxylin and after dehydration mounted in Vitro-Clud (Langenbrinck, Emmendingen, Germany). An experienced pathologist (N.B.) scored the immunohistochemical staining intensity according to the scoring system suggested devised by Remmele and Stegner [16].

### Statistical methods

SPSS version 14.0 (SPSS Inc, Chicago, IL) was used for statistical evaluation. Differences were considered statistically significant at *P *< 0.05. For analysis of the CPA, a Kolmogorov-Smirnov test was applied to test for normal value distribution, followed by a two-sided paired t-test to analyse differences in normal and tumour expression. A statistical association between clinicopathological and molecular parameters was tested, using two-sided Fisher's exact test. Recurrence-free (RFS) and overall survival (OS) were calculated according to the Kaplan-Meier equation.

## Results

### Generation and characterisation of monoclonal anti-RAI3 antibodies

**Figure 1:**
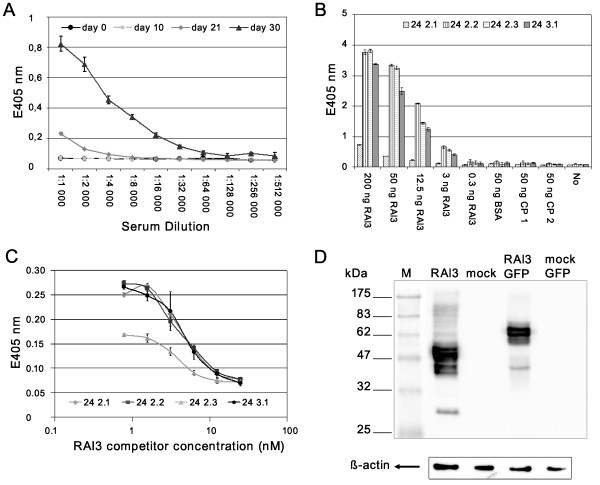
**Characterisation of monoclonal anti-RAI3 antibodies**. **A**:. Direct ELISA of a serial dilution of BALB/c mouse serum carried out at several time points during immunisation. Increasing antbody titre indicates a good humoral immune response. Measurements are from triplicates, expressed as means ± S.D. **B**: Direct ELISA of hybridoma supernatants from four selected monoclones showing a concentration dependent RAI3 signal and no cross-reactivity to control proteins. BSA: bovine serum albumin; CP1: control protein 1, non-related human protein (CD30 ligand); CP2: control protein 2, related human protein, GPCR (GPR30); No: Negative control, no protein coated. Measurements are from triplicates, expressed as means ± S.D. **C**: Competitive ELISA of Mabs pre-incubated with a serial dilution of soluble antigen RAI3. Only unbound antibody can bind to the immobilised antigen on the ELSA plate allowing the comparison and determination of affinities based on the absorption values. Measurements are from triplicates, expressed as means ± S.D. **D**: Western blot analysis of cell extracts from transfected HEK293T cells. Cells expressed either RAI3 (lane 1), mock protein (lane 2), RAI3-GFP (lane 3) or mock-GFP protein (lane 4). Aliquots (10 μg) of total protein from cell extracts are loaded as indicated, with β-actin as the loading control. At least three experiments for Mab 24 2.3 are represented. Equal binding pattern was observed in all experiments.

The human *RAI3 *cDNA was expressed in bacteria and the protein isolated from inclusion bodies and reconstituted into liposomes for immunisation of BALB/c mice. The specific antibody titre during immunisation was monitored by analysing blood samples using a direct ELISA (Figure 1A), allowing the optimum time point for the fusion of spleen cells to be selected. The antibody titre began to increase 21 days after the first immunisation. Between day 21 to day 30, the titre increased from 1: 3000 to 1: 30,000 indicating a good secondary immune response to the recombinant, liposome-integrated protein. Spleenocyte fusion was carried out on day 45, followed by limiting dilution cloning and screening to yield four monoclonal antibodies against RAI3 for further characterisation.

Analysis of hybridoma supernatants by direct ELISA showed that they contained antibodies with high specificity for recombinant RAI3 protein, as revealed by the strong correlation between the amount of coated RAI3 and the absorption signal (Figure 1B). There was no cross reactivity to bovine serum albumin (BSA), the CD30 ligand (an unrelated human protein) or GPR30 (a control human GPCR). Isotyping showed that all four clones produced IgG_1 _antibodies (Table 1) thus prompting our decision to use protein-G affinity chromatography to purify the antibodies from serum-free hybridoma supernatants.

**Table 1:** Monoclonal anti-RAI3 antibodies, overview isotypes and affinity values

Antibody	Isotype	Kd value [nM]
24 2.1	IgG1, kappa	2,2 ± 0,4
24 2.2	IgG1, kappa	1,8 ± 0,9
24 2.3	IgG1, kappa	2,3 ± 0,3
24 3.1	IgG1, kappa	2,7 ± 1,2

**Figure 2:**
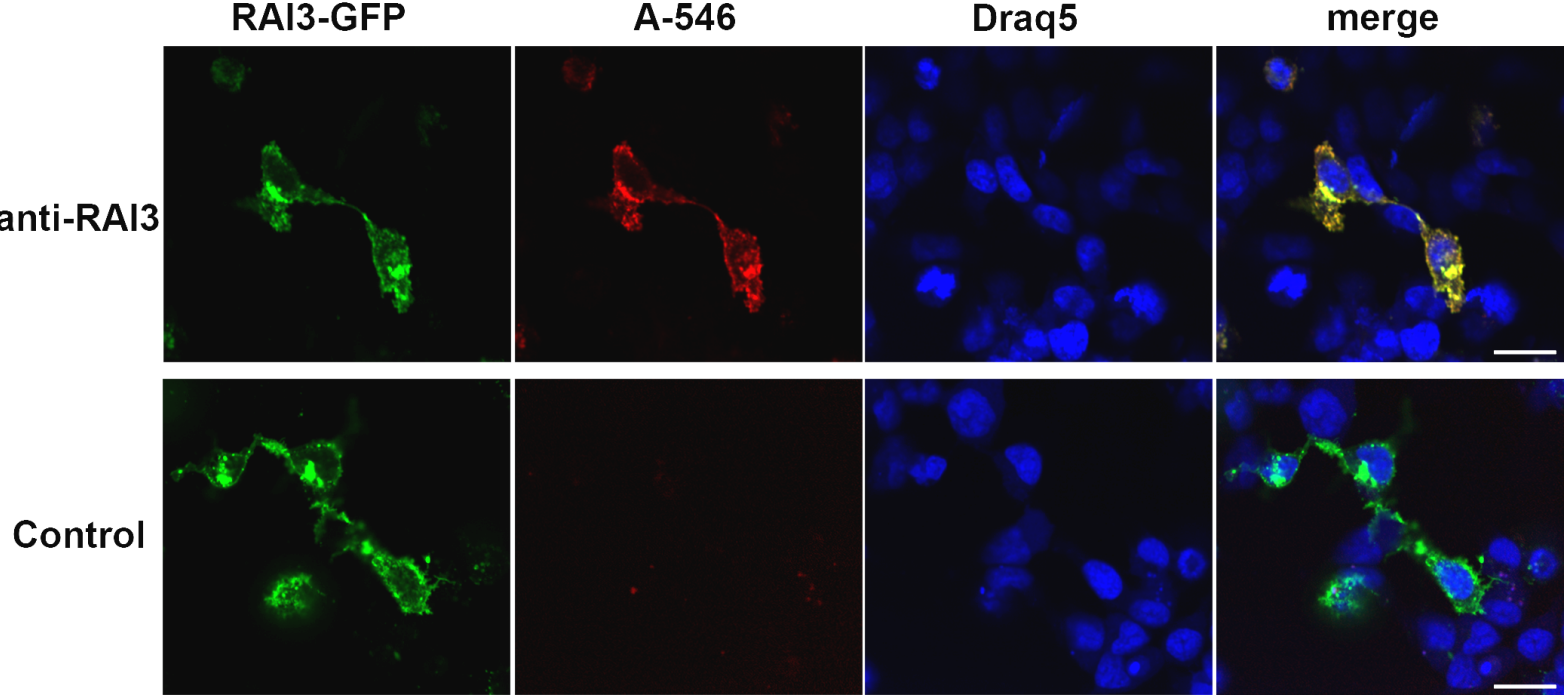
**Immunocytochemistry analysis of HEK293T cells expressing RAI3-GFP**. Transiently transfected HEK293T cells expressing RAI3-GFP were subjected to immunocytochemistry analysis. The upper panels (anti-RAI3) show transfected cells incubated with anti-RAI3 antibody and subsequently with anti mouse AlexaFluor 546-labelled secondary antibody (A546). The immunostaning is pseudo-coloured in red. Co-localisation of the RAI3-GFP and A546 signals is shown in yellow in the merge panel. For the transfected control cells (Control panels) primary anti-RAI3 antibody was omitted in order to exclude unspecific binding of the A546 secondary antibody. Nuclei were stained using Draq5 and are pseudo-coloured in blue. Imaging was carried out by confocal microscopy using a 40× air objective. Scale bar: 20 μm

A competitive ELISA confirmed specific binding of the purified antibodies to recombinant RAI3 in solution (Figure 1C) and was used to calculate binding affinity constants, which were all in the nanomolar range (Table 1). Western blots on lysates from transfected HEK 293T cells confirmed antibody binding to soluble proteins in cells expressing RAI3 and RAI3-GFP but not to soluble proteins in mock-transfected cells (Figure 1D). However, these experiments did not resolve a single distinct band corresponding to the predicted molecular weight of RAI3 (~40 kDa) or its GFP fusion (~67 kDa) despite the general good quality of the lysates demonstrated by the β-actin positive control. This probably reflects aggregation of RAI3, a common problem with hydrophobic proteins, as well as the presence of different glycoforms and perhaps also proteolytic degradation products. Aggregation was also observed in western blots using the purified protein (data not shown). However, specificity becomes clear as these patterns can also be found in lane 3 (GFP fusion) except that the bands in total indicate a protein of a higher molecular weight due to the fusion to GFP. To further confirm RAI3 specificity, we performed immunocytochemistry on HEK293T cells transiently transfected with the expression vector encoding recombinant RAI3-GFP followed by confocal microscopy analysis. The transient transfection produced a heterogeneous population of green fluorescent RAI3-GFP transfected cells and non-fluorescent untransfected cells. This allowed analysis of both cell types within the same cell preparation. Figure 2 (upper panels) clearly shows that the GFP signal of the recombinant receptor and the indirect A546 detection signal of the Mab 24 2.3 anti-RAI3 antibody largely co-localised in transiently transfected HEK293T cells (yellow signal in the merge panel). Interestingly, the non transfected cells did not show any signal which could be ascribed to detection of the endogenous RAI3 receptor by the anti-RAI3 antibody (upper panels of Figure 2). Transfected control HEK293T cells treated with the A546 secondary antibody alone did not display background signals which excluded unspecific binding of the secondary antibody (Figure 2 lower panels). This result confirmed the specificity of the anti-RAI3 antibody in detecting the RAI3 receptor in cells.

Of the four highly-specific antibodies we produced, Mab 24 2.3 was also selected for further immunohistochemical experiments because it showed the cleanest and most intense staining (data not shown).

### Upregulation of RAI3 in primary breast cancer analysed by cDNA dot blot hybridisation

**Figure 3:**
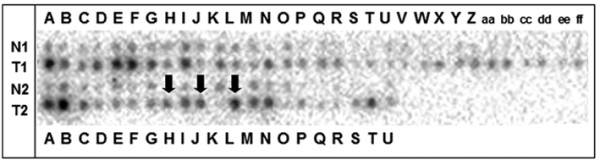
**cDNA dot blot analysis of RAI3 expression in matched normal and t breast tissue**. Expression profiles were determined using cDNA dot blot hybridisation analysis (BD Clontech) containing cDNA pairs derived from 50 matched normal breast tissues (N), tumourous breast tissues (T), and three lymph node metastastic tissues (marked with arrows). Upregulation of *RAI3 *was observed in 30 of 50 primary breast tumours as well as in one of three metastatic lymph nodes, as compared to matched normal breast tissue.

*RAI3 *upregulation was demonstrated by dot blot analysis on a nylon array containing spotted cDNAs derived from 50 matched pairs of normal and tumourous breast tissue (Figure 3). The cancer profiling array showed upregulation (fold change ≥ 2) of *RAI3 *in 30 of 50 primary breast tumours (60%), and in one of three metastatic lymph nodes, compared with matched normal breast tissue (two-sided paired t-test: *P *< 0.001). The mean densitometric intensity in normal tissues was 1600 arbitrary units (standard deviation (SD) ± 818) and 3139 (SD ± 1453) arbitrary units in tumour tissues. Downregulation of *RAI3 *in normal/tumour sample pairs could not be detected. Specificity of the hybridisation probe was demonstrated via Nothern blot and cDNA dot blot controls (see Additional files 1 and 2).

### Expression of RAI3 protein in primary breast carcinomas

**Figure 4:**
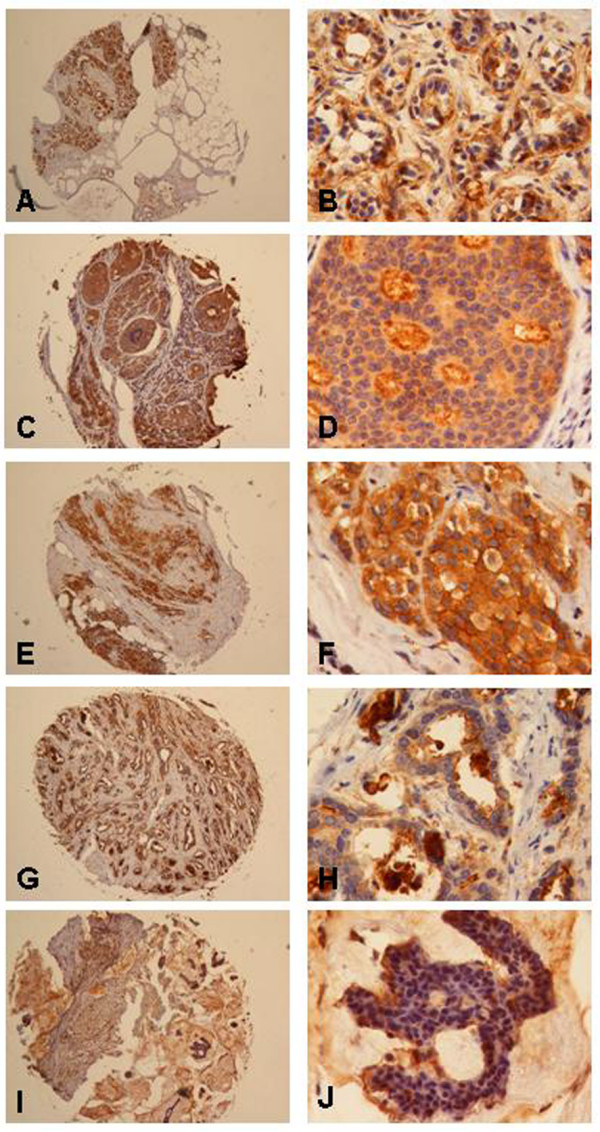
**RAI3 Immunohistochemistry on TMA derived from normal and cancerous breast tissue**. Immunohistochemical expression analysis of RAI3 in normal breast tissue as well as non-invasive and invasive breast tumours using a tissue microarray. (A, B) In normal breast epithelial cells RAI3 expression (IRS = 8) was often weaker than in invasive breast carcinoma cells. (C, D) In ductal carcinoma *in situ *RAI3 expression (IRS = 3) was often less intense compared to invasive breast carcinoma. (E, F) In invasive breast carcinomas (here: ductal type) RAI3 was often expressed more abundantly in the cytoplasm and in the cell membrane (example shows staining with an IRS = 12) than in either ductal carcinoma *in situ *or normal breast tissue. (G, H) In tubular breast carcinomas, RAI3 expression was less abundant (IRS = 3) than in most invasive ductal breast carcinomas. (I, J) In mucinous breast carcinomas, RAI3 expression was also less intense (IRS = 1) in comparison to most invasive ductal breast cancer cells. Magnifications: A, C, E, G, I: 40×; B, D, F, H, J: 400×.

**Figure 5:**
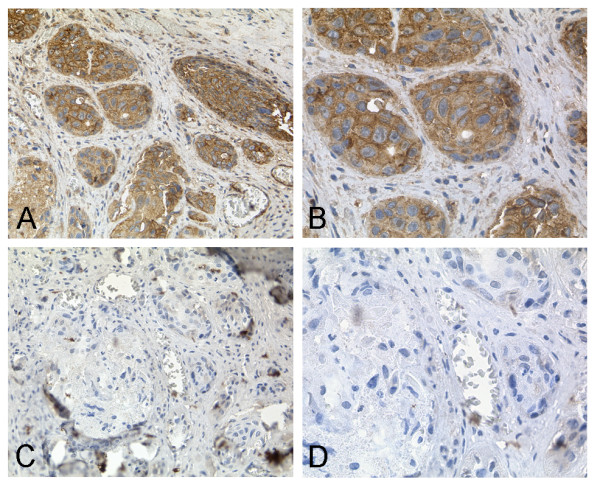
**Competitive RAI3 Immunohistochemistry on cancerous breast tissue**. Immunohistochemical staining of cancerous breast tissue (A, B) in comparison to a competitive immunohistochemical approach (C, D). Strong staining of tumourous tissue was observed using a 1:50 dilution of anti-RAI3 Mab 24 2.3 (A, B). Staining is inhibited by pre-incubation of the antibody with 200-fold molar excess of recombinant RAI3 protein (C, D). Magnifications: A, C: 200×; B, D: 400×.

A large tissue microarray was used to investigate the abundance of cytoplasmic and membrane-bound RAI3 in breast cancer specimens (n = 157) and normal breast tissue specimens (n = 44). Although the median IRS was 4 in both the tumour and normal tissue samples, RAI3 protein levels differed between normal breast tissue and tumours. In normal breast epithelial cells, including myoepithelial cells, RAI3 was often weakly expressed compared to invasive breast carcinoma (Figure 4A and 4B). In ductal carcinoma, *in situ *RAI3 expression was confined to the malignant epithelial cells and was often weaker than in invasive breast carcinoma (Figure 4C and 4D). In invasive ductal breast carcinomas, RAI3 was often generally more abundant in the cytoplasm and in the cell membrane than in either ductal carcinoma *in situ *or normal breast tissue (Figure 4E and 4F, ductal type). In tubular breast carcinomas, a less frequent variant of invasive breast carcinomas with a more favourable prognosis than invasive ductal breast carcinomas, RAI3 protein was expressed less abundantly than in most invasive ductal breast carcinomas (Figure 4G and 4H). In mucinous breast carcinomas, another less frequent variant of invasive breast carcinomas with a more favourable prognosis than invasive ductal breast carcinomas, RAI3 expression was also less abundant in comparison to most invasive ductal breast carcinomas (Figure 4I and 4J). Specificity of the immunohistochemical analysis has been demonstrated by a competitive approach (Figure 5). Staining was greatly reduced by pre-incubating the anti-RAI3 antibody with 200-fold molar excess of recombinant RAI3 protein (Figure 5C, D) compared to staining without competitor (Figure 5A, B).

### Correlation between RAI3 expression and clinicopathological patient parameters

For statistical analysis cytoplasmic/membrane-bound RAI3 protein with an IRS ≥ 5 was considered as 'abundant' whereas an IRS range of 0–4 was considered 'low'. RAI3 expression was not associated with tumour stage, lymph node status, histological grading, focality or histological tumour type (Table 2). Furthermore, RAI3 was neither associated with the presence of the oestrogen receptor (*P *= 0.557) nor the progesterone receptor (*P *= 0.765). Finally, we observed no association between RAI3 expression and high expression of the HER2 receptor as determined by immunohistochemistry (DAKO score 3+; *P *= 0.638) (Table 2).

**Table 2:** Clinicopathological and immunohistochemical parameters in relation to RAI-3 immunoreactivity

Variable	Categorisation	RAI-3 immunoreactivity			
		n analysable	low^b^	abundant^b^	p^c^
***Clinicopathological data:***					
Tumour stage^a^					
	pT1	46	29	17	0.134
	pT2	74	31	43	
	pT3	12	5	7	
	pT4	24	13	11	
Lymph node status^a^					
	pN0	68	34	34	0.713
	pN1-3	83	44	39	
Histological grade					
	G1	16	9	7	0.572
	G2	67	36	31	
	G3	72	33	39	
Multifocality					
	unifocal tumour	130	69	61	0.086
	multifocal tumour	26	9	17	
Histological type					
	ductal	130	66	64	0.905
	lobular	11	5	6	
	other	13	6	7	
					
***Immunohistochemistry (IHC):***					
Oestrogen receptor status					
	negative (IRS 0–2)	41	23	18	0.557
	positive (IRS 3–12)	89	45	44	
Progesterone receptor status					
	negative (IRS 0–2)	95	46	49	0.765
	positive (IRS 3–12)	41	21	20	
HER2 status					
	weak (0–2+)	109	51	58	0.638
	strong (3+)	25	13	12	

^a^According to UICC: TNM Classification of Malignant Tumours. 6^th ^edn (2002) Sobin LH, Wittekind CH (eds) Wiley: New York

^b^RAI-3 immunoreactivity: low = IRS 0–4, abundant = IRS 5–12

^c^Pearson test (two-sided), bold face representing significant data (*P *< 0.05)

### Prognostic impact of RAI3 expression

To investigate the impact of RAI3 overexpression on patients' clinical outcome, we calculated univariate survival probability curves with respect to the immunohistochemistry results. We found that abundant RAI3 expression in breast cancer (IRS ≥ 5) was not associated with significant alterations in the outcome regarding overall (*P *= 0.816) and recurrence-free (*P *= 0.897) survival as shown by Kaplan-Meier analysis (Table 3). The other factors established in clinical routine, including tumour stage, lymph node status, histological grading as well as the status of the hormone receptors and HER2, show significant correlations in overall and/or recurrence-free survival analysis, underlining the fact that the set of breast carcinomas used in our investigation was not biased (Table 3).

**Table 3:** Univariate analysis of factors regardingoverall survival (OS) and recurrence-free survival (RFS)

Variable	Categorisation	Tumour-related death (OS)			Tumour recurrence (RFS)		
		n	events	p^c^	n	events	p^c^
***Clinicopathological data:***							
Tumour stage^a^							
	pT1	46	7	**<0.0001**	44	8	**<0.0001**
	pT2	74	27		71	32	
	pT3	12	3		11	4	
	pT4	24	16		21	13	
Lymph node status^a^							
	pN0	68	9	**<0.0001**	66	13	**<0.0001**
	pN1-3	83	40		79	42	
Histological grade							
	G1	16	4	**0.005**	15	5	**<0.0001**
	G2	67	16		62	15	
	G3	72	33		70	37	
Multifocality							
	unifocal tumour	130	43	0.545	123	47	0.689
	multifocal tumour	26	10		24	10	
Histological type							
	ductal	130	44	0.787	126	52	0.432
	lobular	11	5		9	2	
	other	13	4		11	3	
							
***Immunohistochemistry (IHC):***							
Oestrogen receptor status							
	negative (IRS 0–2)	41	17	0.094	41	21	**0.034**
	positive (IRS 3–12)	89	26		84	26	
Progesterone receptor status							
	negative (IRS 0–2)	95	43	**<0.0001**	88	43	**0.001**
	positive (IRS 3–12)	41	5		41	6	
HER2 IHC							
	weak (0–2+)	109	32	**0.006**	101	37	0.128
	strong (3+)	25	14		25	12	
RAI-3^b^							
	low (IRS 0–4)	78	25	0.816	76	29	0.897
	abundant (IRS 5–12)	78	28		71	28	

^a^According to UICC: TNM Classification of Malignant Tumours. 6^th ^edn (2002) Sobin LH, Wittekind CH (eds) Wiley: New York

^b^RAI-3 immunoreactivity: low = IRS 0–4, abundant = IRS 5–12

^c^Log-rank test (two-sided), bold face representing significant data (*P *< 0.05)

## Discussion

We generated four monoclonal antibodies against the human GPCR RAI3 and carried out a systematic analysis of RAI3 expression in human breast cancer. Members of the GPCR superfamily are known to be involved in the regulation of many physiological processes including cell growth and differentiation, and are therefore regarded by the pharmaceutical industry as interesting therapeutic targets [1,17,18]. In terms of antibody generation, GPCRs in general are often considered as difficult antigens since the expression and purification of the recombinant protein is a challenging approach that limits the availability of the protein [10,19,20]. We have overcome this challenge by combining recombinant protein expression in bacteria with a technique for reformulating the RAI3 protein in liposomes prior to immunisation, allowing the generation of monoclonal antibodies using standard hybridoma technology. Only polyclonal antibodies against RAI3 are commercially available, so our work represents an important step forward, i.e. the first monoclonal anti-RAI3 antibodies. Monoclonal antibodies are more suitable as therapeutics because nonspecific cross-reactions are less likely to occur. Furthermore, the quality of polyclonal antibodies may vary considerably between batches whereas monoclonal antibodies, which originate from a single cell, are more consistent. Among four independent monoclonal antibodies generated by hybridoma technology, we selected Mab 24 2.3 for further immunohistochemical experiments as it provided the clearest signals and was able to detect RAI3 in formaldehyde-fixed, paraffin-embedded tissues which makes it easily applicable in the clinicopathological routine.

To determine whether Mab 24 2.3 was useful for the characterisation of human breast carcinomas, we carried out a systematic analysis of breast cancer and normal breast tissue on a tissue microarray. Previous studies have shown that *RAI3 *mRNA is upregulated in several breast cancer tissues and cell lines [5,7,9] but there has been no similar study at the protein level because of the lack of useful reagents and a immunohistochemical characterisation of the RAI3 protein in normal and breast cancer tissue has not been published. The systemic analysis of RAI3 protein expression in human breast cancer tissue is interesting because the protein has been proposed as membrane-bound [3], making it a potential therapeutic target as well as a useful biomarker. The most prominent therapeutic monoclonal antibody is Herceptin^® ^(trastuzumab) which is indicated for breast cancers involving the upregulation of HER2, a receptor tyrosine kinase belonging to the epidermal growth factor receptor (EGFR)/HER family [21]. Upregulation of HER2 is observed in 10–34% of invasive breast tumours and offers a poor prognosis. The HER2 status of a breast cancer patient is therefore a very important clinical indicator used to select a therapeutic strategy, and HER2-positive breast tumours are often treated with Herceptin^®^. Nevertheless, most patients develop resistance to Herceptin during therapy [22], so the discovery of additional biomarkers and potential therapeutic targets is of great interest, since this provides scope for novel or combined therapies in Herceptin-resistant tumours. RAI3 may be such a candidate molecule and monoclonal antibodies against RAI3 could be used in diagnosis and in the treatment of breast cancer. However, because the function of RAI3 in normal and malignant cells is poorly understood and controversial [5,6,8], a thorough analysis of its expression in human breast cancer tissue is necessary prior to further development. Expression of RAI3 in human cancerous and normal breast tissue has not been analysed by immunohistochemistry, thus far. In the current study, a systematic characterisation of RAI3 expression in human cancerous and normal breast tissue, both at the cDNA and the protein level, is here provided for the first time.

In a previous study [6], *RAI3 *mRNA levels were measured by quantitative RT-PCR in the normal mammary cell lines HMEC 50 and HMEC 21 as well as in the malignant breast epithelial cell lines MDA-MB-468, BT-20, BT-549, SK-BR-3, T47D, MCF7, ZR-75-1 and BT474. Upregulation of *RAI3 *mRNA (50%) was observed in the oestrogen receptor-negative breast cancer cell lines MDA-MB-468, BT-20, BT-549, SK-BR-3 compared to the normal mammary cell lines HMEC 50 and HMEC 21. However, we could not detect a significant correlation between RAI3 expression and the hormone receptor status in our analysis. Furthermore, similar results indicating RAI3 upregulation were obtained in a small set of lung, colon and pancreas cell lines, compared to one sample originating from normal tissue [6]. The overexpression of RAI3 in human breast cancer was also shown by Nagahata *et al. *[5,9]. Quantitative RT-PCR also showed that RAI3 levels were higher in 19 out of 25 primary breast cancers compared to matched normal tissues and in 6 of 11 breast cancer cell lines and in HEK293 cells [5]. In our study, we confirmed *RAI3 *mRNA overexpression by cDNA dot blot analysis, showing *RAI3 *upregulation in 60% of a large collection of 50 matched human breast carcinomas compared to the corresponding normal breast tissues. However, we did not observe similar upregulation at the protein level based on immunohistochemical analysis of a TMA. Here we analysed a large cohort of 157 breast cancer specimens and 44 normal breast tissue specimens that were not matched. The median IRSs for normal and cancerous breast tissues showed no statistically significant difference. A possible explanation for the discrepancy between the mRNA and protein results may be the presence of larger amounts of connective tissue in normal tissue samples compared with the corresponding tumour tissue samples. Thus, RNA preparations from such samples would contain an excess of mRNA originating from RAI3-negative connective tissue, resulting in lower overall signal levels compared to the tumour samples, a phenomenon that could also have affected previous RNA-based studies of breast cancer. In an immunohistochemical approach like the TMA, only normal and cancerous epithelial cells are compared directly, so the presence of connective tissue does not influence the result. Furthermore, we cannot exclude the possibility of posttranscriptional regulation of the RAI3 gene. In such a case, translation of mRNA into protein may be inhibited which would result in discordant mRNA and proteins levels [23]. This could also explain the fact that we could not detect endogenous RAI3 in HEK 293T by western blot and immunocytochemistry analysis of untransfected cells (see Additional file 3). Another explanation for this discrepancy may be clonal differences in the used cell lines from different laboratories. There is a tremendous number of different factors that could account for changes of the physiological status of cells and can affect the expression levels of certain proteins, e.g. age of the cells, numbers of passages, cell culture medium, supplements, etc. [24]. In addition, it should be noted that *RAI3 *expression data derived from cells cultured *in vitro *is liable to misinterpretation because *RAI3 *is induced in cancer cells by the presence of serum in the culture medium. This could introduce a bias if normal cells are cultivated under different conditions, e.g. without serum [25]. However, such factors can be excluded from our analysis because we used tissue samples rather than cell lines.

A cDNA microarray and RT-PCR study of 20 oestrogen receptor-negative breast cancers identified *RAI3 *as one of the genes indicating poor patient survival (*RAI3 *was upregulated in the group of 10 patients who had died of breast cancer within 5 years after surgery [9]). Genuine *RAI3 *upregulation has to be questioned as the authors used a very small collection of breast cancer specimens. In our study, using a TMA containing a much larger set of breast carcinomas, we found no significant correlation between *RAI3 *expression and overall or recurrence-free survival.

The ambiguous role of RAI3 in oncogenesis is underlined by studies that indicate *RAI3 *is a tumour suppressor gene in lung cancer, based on increased lung tumour prevalence in *RAI3 *knock-out mice [8]. Additionally, *RAI3 *mRNA levels were reduced in 11 of 18 human lung cancer samples compared to adjacent normal tissue samples. In our study, we found no evidence in either the CPA or TMA experiments of *RAI3 *downregulation in human breast carcinoma. The RAI3 protein was distributed heterogeneously throughout the normal breast tissue and tumour samples. Therefore, whether RAI3 could be a target for future breast cancer therapy is still unclear. Further studies in larger cohorts of matched normal breast and tumour tissues may provide more detailed information about the upregulation of *RAI3 *in breast tumours because the induction of RAI3 was demonstrated in the CPA, which comprised matched tissue samples. Further functional studies are also required to determine the receptor's mode of action and its ligand.

In summary, the novel monoclonal anti-RAI3 antibody Mab 24 2.3 may be useful in further studies to determine the potential of RAI3 as a tumour marker. It would be particularly beneficial to study the expression of the RAI3 protein in large TMAs representing a range of different tissues to gain better insight into the role of RAI3 in human cancer and to evaluate its potential as a target for antibody-based cancer therapy.

## Conclusion

We generated a highly specific anti-RAI3 monoclonal antibody (Mab 24 2.3) which could bind the RAI3 antigen in cell lysates, cells in culture and paraffin-embedded tissues, making it a useful clinical diagnostic tool. Immunohistochemical analysis of normal human breast tissues and tumours using Mab 24 2.3 showed widespread expression of the RAI3 protein on a tissue microarray of human breast carcinomas. Furthermore, we demonstrated upregulation of *RAI3 *mRNA in breast tumours using cDNA dot blot analysis. RAI3 is a potential novel biomarker for breast cancer that might be useful as a therapeutic target due to its membrane localisation and its association with cancer cell proliferation. However, we were unable to establish a correlation between patient survival and RAI3 expression. Therefore, further analysis of the protein is required using larger cohorts representing a variety of tissues, to reveal the functional role of RAI3 expression in human cancer.

## Competing interests

The authors declare that they have no competing interests.

## Authors' contributions

HJ: participated in study design, data interpretation, establishment and evaluation of antibody generation and characterisation, assisted in immunocytochemistry and immunohistochemistry and drafted the manuscript. NB: participated in study design, data analysis, data interpretation, establishment and evaluation of immunohistochemistry and drafted the manuscript. ED: participated in study design and coordination, molecular and data analysis, data interpretation and drafting of the manuscript. AH: provided the tissue microarray and participated in data analysis and immunohistochemical evaluation. AtH: participated in the establishment of the immunohistochemistry and data analysis. SdF: participated in data analysis of immunocytochemistry and confocal laser scanning microscopy. HK and AT: collaborated in the study design, produced and provided the antigen and contributed parts of the manuscript. SB: conceived of the study and participated in its design and coordination and critically revised the manuscript. TK: conceived of the study, and participated in design and coordination of the study, data analysis and interpretation and helped to draft the manuscript. All authors read and approved the final manuscript.

## Pre-publication history

The pre-publication history for this paper can be accessed here:

http://www.biomedcentral.com/1471-2407/9/200/prepub

## Supplementary Material

Additional file 1 **RAI3 Nothern-blot of total RNA**. Northern Blot using the Clontech Multiple Tissue Northern Blot (MTN) containing poly A+ RNA derived from human heart, brain, placenta, lung, liver, skeletal muscle, kidney and pancreas. This blot detects two known RAI3 transcripts of 6.8 and 2.4 kb in size and demonstrates extraordinary abundant *RAI3 *expression in human lung tissue. Click here for file

Additional file 2 **RAI3 cDNA controls of cancer profiling array**. Complete Cancer Profiling Array with negative controls (and cell lines) that are positioned at the right side of the nylon membrane. This analysis shows that yeast total RNA, yeast tRNA, E. coli DNA, poly A+ RNA and ubiquitin cDNA do not exhibit a cross-hybridisation signal. Genomic DNA of course contains the *RAI3 *gene, therefore a weak hybridisation signal can be expected. Finally the probe was hybridised to an internal positive control (spotted RAI3-cDNA) and was able to detect less than 10 pg of RAI3 cDNA. Click here for file

Additional file 3 **Western blot analysis of breast cancer cell lines MCF-7 and MDA-MB-453**. Western blots of lysates from RAI3-transfected (RIII) and RAI3-GFP-transfected (RGFP) HEK293T cells, in comparison to HEK293Twt cells, and breast cancer cell lines MCF-7 and MDA-MB-453 (MB453). Detection with anti-RAI3 Mab 24 2.3, HRPO-labelled anti-mouse secondary antibody and ECL as substrate. Endogenous RAI3 cannot be detected in HEK293T wt cells. However, in RAI3-positive MCF-7 cells low levels of RAI3 can be detected in western blot using anti-RAI3 antibody. As negative control cell line MDA-MB-453 are used that are reported as RAI3-negative. Click here for file
