# Supplementary figures and images for: Production and characterisation of monoclonal antibodies against RAI3 and its expression in human breast cancer
Source: BMC Cancer. 2009 Jun 24;9:200. doi: 10.1186/1471-2407-9-200 (PMC2711971; doi:10.1186/1471-2407-9-200)

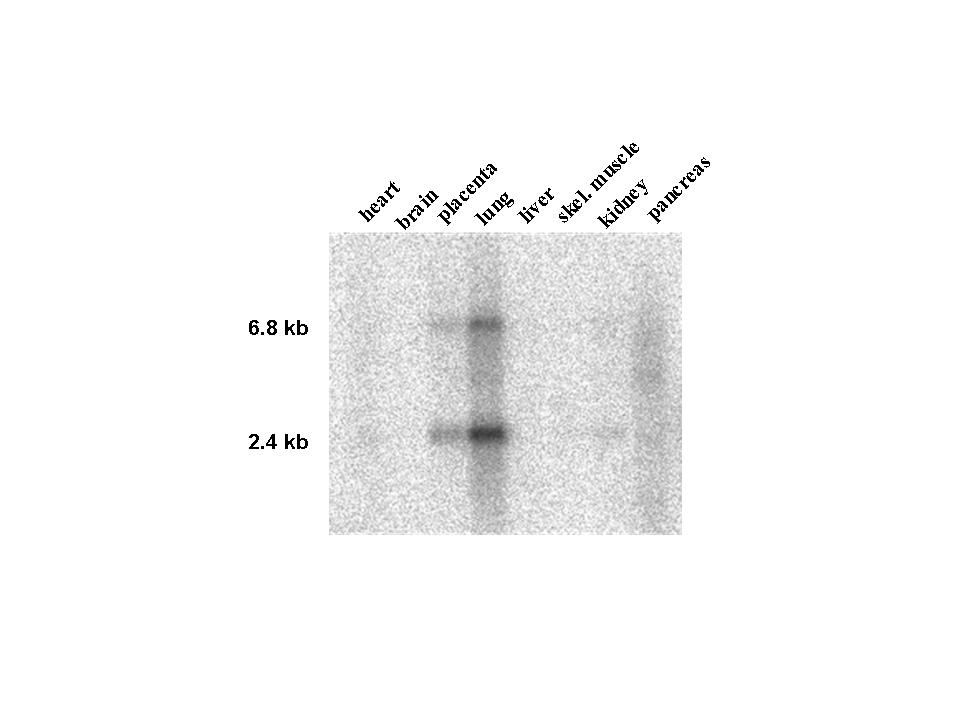

Supplement: Additional file 1 — RAI3 Nothern-blot of total RNA. Northern Blot using the Clontech Multiple Tissue Northern Blot (MTN) containing poly A+ RNA derived from human heart, brain, placenta, lung, liver, skeletal muscle, kidney and pancreas. This blot detects two known RAI3 transcripts of 6.8 and 2.4 kb in size and demonstrates extraordinary abundant RAI3 expression in human lung tissue. [file 1471-2407-9-200-S1.jpeg]

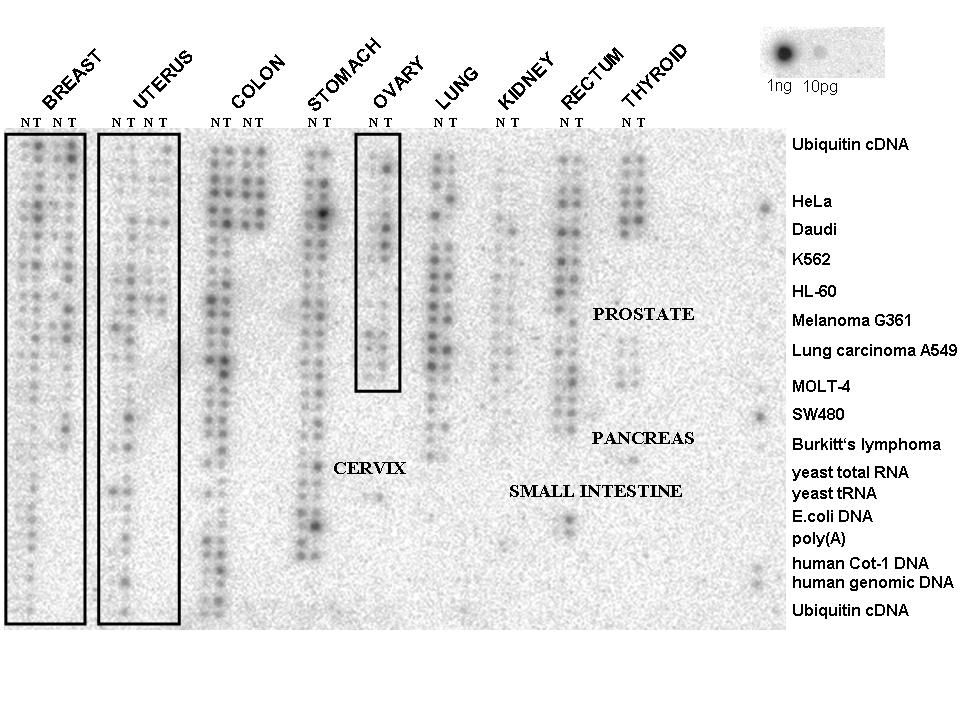

Supplement: Additional file 2 — RAI3 cDNA controls of cancer profiling array. Complete Cancer Profiling Array with negative controls (and cell lines) that are positioned at the right side of the nylon membrane. This analysis shows that yeast total RNA, yeast tRNA, E. coli DNA, poly A+ RNA and ubiquitin cDNA do not exhibit a cross-hybridisation signal. Genomic DNA of course contains the RAI3 gene, therefore a weak hybridisation signal can be expected. Finally the probe was hybridised to an internal positive control (spotted RAI3-cDNA) and was able to detect less than 10 pg of RAI3 cDNA. [file 1471-2407-9-200-S2.jpeg]

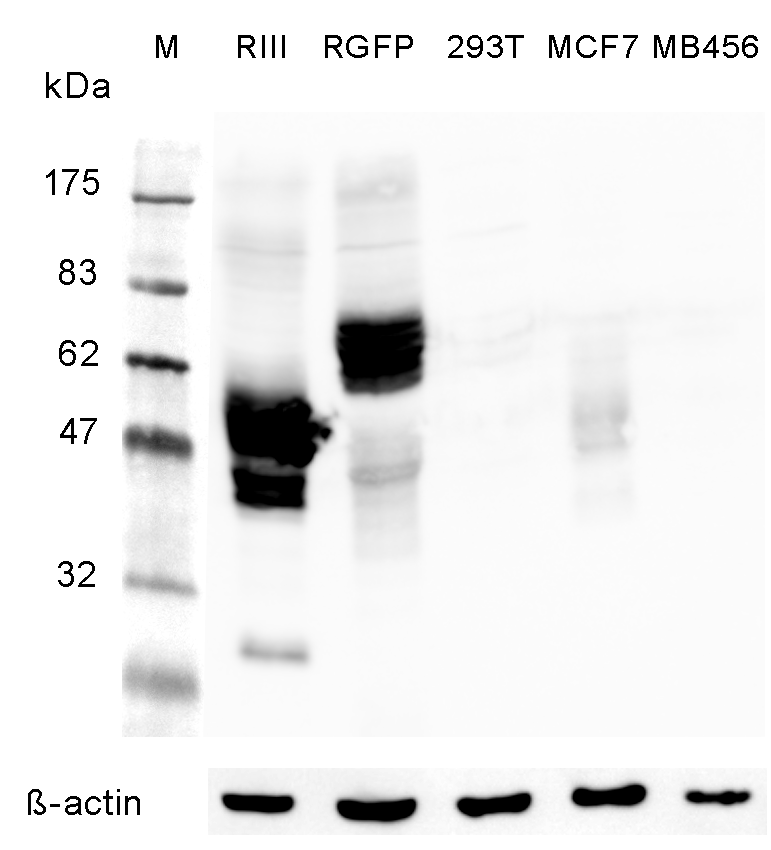

Supplement: Additional file 3 — Western blot analysis of breast cancer cell lines MCF-7 and MDA-MB-453. Western blots of lysates from RAI3-transfected (RIII) and RAI3-GFP-transfected (RGFP) HEK293T cells, in comparison to HEK293Twt cells, and breast cancer cell lines MCF-7 and MDA-MB-453 (MB453). Detection with anti-RAI3 Mab 24 2.3, HRPO-labelled anti-mouse secondary antibody and ECL as substrate. Endogenous RAI3 cannot be detected in HEK293T wt cells. However, in RAI3-positive MCF-7 cells low levels of RAI3 can be detected in western blot using anti-RAI3 antibody. As negative control cell line MDA-MB-453 are used that are reported as RAI3-negative. [file 1471-2407-9-200-S3.tiff]
